# Voice quality preservation in thyroid surgery with neuromonitoring

**DOI:** 10.1007/s12020-018-1614-4

**Published:** 2018-05-05

**Authors:** Beata Wojtczak, Krzysztof Sutkowski, Krzysztof Kaliszewski, Zdzisław Forkasiewicz, Bartłomiej Knychalski, Michał Aporowicz, Marek Bolanowski, Marcin Barczyński

**Affiliations:** 1 Department and Clinic of General, Gastroenterological and Endocrine Surgery, Wroclaw Medical University, Wroclaw, Poland; 2 Department of Endocrinology, Diabetes and Isotope Therapy, Wroclaw Medical University, Wroclaw, Poland; 3 Department of Endocrine Surgery, Third Chair of General Surgery, Jagiellonian University Medical College, Kraków, Poland

**Keywords:** Thyroid, Surgery, Neuromonitoring, Voice, Quality of life

## Abstract

**Purpose:**

Voice problems are common after thyroidectomy. The aim of this study was to assess the voice related quality of life after thyroidectomy with neuromonitoring. The sociodemographic and treatment factors influencing the quality of voice after the operation were investigated.

**Methods:**

A total of 40 patients after thyroidectomy with neuromonitoring were enrolled into the study. The voice outcome was analyzed pre and postoperatively by two validated self-assessment questionnaires: Voice Handicap Index and Voice-Related Quality of Life survey.

**Results:**

All external branches of the superior laryngeal nerve were identified during the operation. There were no recurrent laryngeal nerve palsies. The mean total VHIs before and after thyroid operation were 1.2 [SD 2.564] and 2.8 [SD 6.944], respectively (*p* = 0.5). Preoperatively, the mean overall score for the V-RQOL was 99.6; postoperatively 98.7 (*p* = 0.05). A strong correlation between the V-score of the V-RQOL and O-score of the VHI before and after thyroidectomy was observed (both *p* < 0.001).

There was no correlation between V-RQOL or VHI and sex, the kind of thyroid operations, diagnosis, thyroid function, the mean volume of the goitre, the presence of retrosternal position and the extent of thyroid operations (*p* > 0.05). A small correlation between the mean age of the patients and postoperative O-Score of the VHI (*p* = 0.007650) and between the mean age and postoperative V-Score for the V-RQOL (*p* = 0.00648) was observed.

**Conclusions:**

The use of neuromonitoring in thyroid surgery is beneficial for patients to improve voice quality. The identification and preservation of EBSLNs is crucial to eliminate altered voice after thyroidectomy.

## Introduction

There are many indications for surgical treatment of the thyroid gland. Thyroidectomy is usually recommended in multinodular goitre with compressive symptoms, dyspnoea or dysphagia, or in Graves’ disease with orbitopathy and is a treatment of choice in malignancy or suspicious findings on fine needle aspiration biopsy (FNAB).

In recent years, an increase in the rate of thyroid surgery has been observed. For example, in the last three decades, the rate of thyroid operations has tripled in the United States [1]. Moreover, more operations are performed due to thyroid cancer, thanks to better access to ultrasound examinations and FNAB. These examinations have increased the identification of malignancy in nodules from 15 to 50%, particularly in small papillary cancers [2]. The extent of surgery (total vs. subtotal) has been controversial until recently [3], but currently, total thyroidectomy is recommended in most high-volume thyroid surgery units [4, 5]. A perfectly performed total thyroidectomy is based on complete thyroid mass removal, with central neck dissection in case of thyroid cancer, with preservation of parathyroid gland function and anatomical course and function of recurrent laryngeal nerves. However, thyroid operations are still associated with complications and the impact of these surgeries on the voice determines the quality of life after them [6]. There are many causes of thyroidectomy-related voice changes. The most common are injuries of the recurrent laryngeal nerve (RLN) and external branch of the superior laryngeal nerve (EBSLN). In some patients, the voice after thyroid surgery is changed by cricothyroid muscle injury, strap muscle injury or local haematoma, or intubation-related injury can happen [6]. The consequences of voice-related changes after thyroid surgery constitute an impairment of the quality of life. In the case of RLN injuries, patients claim a hoarse voice, breathy voice or vocal fatigue. Injury of the EBSLN either reduces the ability to produce high frequency tones or causes a hoarse and breathy voice [7, 8]. It is difficult to estimate the true number of vocal complications, because not all patients after thyroid surgery have undergone laryngoscopy [9]. According to a systemic review of 27 articles (25,000 patients after thyroid operations), the incidence of temporary vocal fold palsy was 9.8% and the incidence of permanent RLN palsy was 2.3% [10]. The prevalence of EBSLN injury is much more difficult to assess due to limited data and difficult laryngeal examinations to confirm these complications. The only definitive way to diagnose EBSLN injury is cricothyroid muscle electromyography. In the literature we find that the prevalence of this injury varies from 0 to 58%. These complications are the most commonly underestimated morbidity after thyroidectomy [8]. Moreover, voice changes may occur after thyroidectomy without evident laryngeal nerve injury [6]. Thus, patients should be made aware of possible mild changes in the voice after thyroid surgery without any recurrent laryngeal nerve complications. Taking into account the fact that the voice is an essential part of human communication, it especially should be taken care of pre-, intra- and postoperatively, according to the clinical guidelines by Chandrasekhar et al. [6]. An evaluation of the voice quality of life after thyroid surgery gives surgeons some useful information about the quality of treatment.

There are some publications about the voice quality of life after thyroid surgery, but none of them takes into account the use of intraoperative neuromonitoring (IONM) during thyroidectomy. In recent years, IONM has been standardized and has gained acceptance among surgeons during thyroid operations. Neuromonitoring has broadened our knowledge on RLN anatomy, facilitated routine RLN identification and positively influenced the rate of RLN and EBSLN injuries. Currently, there is no doubt that IONM should be used in difficult thyroid surgery, in particular in redo surgery, thyroid cancers and Graves’ disease operations, where the risk of RLN injury is extremely high [7, 8].

The aim of this study was to assess the voice-related quality of life after thyroid surgery with the utilization of neuromonitoring.

## Material and methods

This prospective clinical study included 40 patients, who underwent thyroid operations with the utilization of IONM at the Department of General, Gastroenterological and Endocrine Surgery at Wroclaw Medical University between January 2015 and July 2017.

Before surgical treatment, all patients were precisely diagnosed and treated by a general practitioner or endocrinologist. All patients were euthyroid; TSH (thyroid-stimulating hormone) and fT4 (free thyroid hormones) were measured before the operations. In the case of hyperthyroidism before the operations, the patients were treated with antithyroid drugs and/or in the case of Graves’ disease, radioiodine therapy was used. We did not give Lugol’s liquid to patients with hyperthyroidism in the process of preparation. In all patients, before thyroid surgery ultrasonography examinations and chest X-ray were performed. FNAB was performed to find any suspicious tissues in the nodular goitre. All patients received informed consent prior to surgical treatment with information on possible complications related to both RLN and EBSLN injury.

Patients enrolled into the study were operated on with IONM. The Nerve Integrity Monitoring System (NIM-3, Medtronic Xomed, Jacksonville, FL, USA) with endotracheal Flex tubes size 7.0 or 7.5 was used in the study. To test the nerves, intermittent neuromonitoring was used. The nerves were stimulated by a single-use, monopolar stimulator probe, set at 1 mA to 2 mA, impulse duration at 100 μs and frequency at 4 Hz. The monitor was set for an event threshold of 100 μV. Both RLNs and EBSLNs were monitored with the approach recommended by the International Neural Monitoring Study Group [7, 8]. The standard of IONM was carried out using a typical 6-step procedure: L1, V1, R1, R2, V2, L2, which means that all patients before and after surgery underwent laryngoscopy; vagal and recurrent laryngeal nerves were evaluated before and after removing the goitre. In the case of EBSLN monitoring, CTM (cricothyroid muscle) twitch was evaluated. For both RLN and EBSLN, positive signal/stimulation was recognizable when the electromyographic waveform was higher than 100 μV.

All thyroid operations were performed by a single high-volume thyroid surgeon (B.W.), who performs more than 100 thyroid operations per year.

Patients enrolled into the study underwent preoperative direct laryngoscopy. All of them had correct vocal cord function. Vocal cord palsy recognized preoperatively was the exclusion criteria. All patients after thyroid operations had direct laryngoscopy by an independent laryngologist.

In order to evaluate the quality of voice, the surveys were completed by all patients before and about 2 months after the operation.

Voice-Related Quality of Life (V-RQOL) Survey shows how a voice problem could interfere with daily activities. There are ten questions about problems related to the voice in the survey which evaluates how severe this problem is and how frequently it happens using a 1–5 scale (5 representing the most serious situation). The answers are based upon average voice quality for the past 2 weeks. By adding all of the points, the patient’s own voice-related quality of life (V-RQOL) can be estimated. The scale ranges from 0 to 100, with 100 being the best result—'excellent', 75—'fair to good', 50—'poor to fair' and 25—'poor'. The V-RQOL was adapted from the 'V-RQOL Measure' from the Vocal Health Center at the University of Michigan Health System. The 10-item V-RQOL measure performs well in tests of reliability, validity, and responsiveness, and it carries a low burden [11]. In the study, the Polish version of the V-RQOL was used, which is easy to perform in clinical practice [12]. To sum up, the survey scoring algorithm for V-RQOL measure was used [11].

Voice Handicap Index (VHI) was developed and validated in 1997 by Jacobson [13]. It is a 30-item self-administered questionnaire to quantify the patient’s perception of disability resulting from voice disorders. This survey consists of three parts, the functional, physical and emotional aspects of voice disorders. The functional (F-VHI) subscale refers to patient communication problems, the emotional (E-VHI) subscale describes patient affective responses to voice disorder and the physical (P-VHI) one relates to patient perception of his or her voice. All the questions are administered by patients themselves regardless of the type of their voice disorder in a five-point scale manner for each item (from 0 = never to 4 = always). Patients with severe voice disorder would gain higher total VHI scores (maximum 120). A VHI score between 0–30 reflects a minimal/mild VHI, 31–60 a moderate VHI, and between 61 and 120 a severe voice handicap. It is a valid and reliable instrument for assessing the self-perception of a patient’s voice handicap [14]. In the study, the Polish version of the voice handicap index was used [15].

The Bioethics Committee of Wroclaw Medical University in Poland approved the protocol of this study.

The data were entered into a Microsoft Office Excel 2007 (Microsoft Corp) worksheet, and statistical analysis was performed using the Statistical Package R 3.2.2 (The R Foundation for Statistical Computing, Auckland, New Zealand). The data from the V-RQOL and VHI scales were correlated with sociodemographic characteristics (age, sex) and treatment-related variables (diagnosis, thyroid function category, goitre volume and surgery category) as well as with each other using the Spearman rho, U Mann–Whitney and binomial nonparametric tests. A p value less than 0.05 was considered significant.

## Results

### Patients’ sociodemographic and treatment details

The completed questionnaires were received from 40 patients who underwent thyroid operation with the application of IONM. All questionnaires were completed before and in the second month after the operation. There were 30 women and 10 men with a mean age of 53.8 years (range: 18–74). Among them 35 (87.5%) underwent primary thyroid operations and 5 (12.5%) were reoperated on due to the recurrence of multinodular goitre many years after the first operation. The indications for thyroid operations were: multinodular goitre—26 (65%), toxic multinodular goitre—3 (7.5%), Graves’ disease—3 (7.5%), thyroid carcinoma—8 (20%). The following thyroid operations were performed: 3 (7.5%) lobectomies, 29 (72.5%) thyroidectomies, 8 (20%) thyroidectomies with central lymph node clearance (level VI). All detailed demographic and intraoperative data are shown in Table 1.

**Table 1:** Demographic characteristics and intraoperative information of the patients enrolled in the study

Patients, number	40 (100%)
Recurrent laryngeal nerves at risk, number	77
Mean age (range)	53.8 (18–74)
Female, number	30 (75%)
Male, number	10 (15%)
Primary operations, number	35 (87.5%)
Reoperations, number	5 (12.5%)
Volume of the goitre mean, range (ml)	51.325 (12–146)
Retrosternal goitre, number	27 (67.5%)
*Benign disorders*—32 (80%)	
Nodular goitre, number	26 (65%)
Toxic nodular goitre, number	3 (7.5%)
Graves’ disease, number	3 (7.5%)
*Thyroid carcinoma*—8 (20%)	
Papillary thyroid carcinoma, number	7 (17.5%)
Poorly differentiated thyroid carcinoma, number	1 (2.5%)
*Thyroid operations*	
Lobectomy, number	3 (7.5%)
Thyroidectomy, number	29 (72.5%)
Thyroidectomy with lymphadenectomy, number	8 (20%)

### Thyroid operation with neuromonitoring

All RLNs and EBSLNs were identified in all patients. In one patient, transient loss of signal was observed during the operation with the recovery of the signal within 10 min. The proper movement of vocal cords before and after thyroid operations was confirmed in all patients. In two patients, postoperative hypoparathyroidism was treated with calcium intravenous supplementation. There were no bleeding complications after the thyroid surgery.

### VHI scale

The mean total score (T-VHI) before and after thyroid operation were divided into individual domains: mean functional score (F-VHI), a physical score (P-VHI) and an emotional (E-VHI) score and were presented in Table 2. The binomial test revealed that the null hypothesis cannot be rejected, which means that the number of the patients who got VHI score = 100 (and <100 respectively) was not significantly different before and after the treatment. It is shown in Table 2.

**Table 2:** Results of descriptive statistical analysis for voice self-assessment variables before and after thyroidectomy

		Before thyroid operation				After thyroid operation				
	Number of patients	Min	Max	Mean	SD	Min	Max	Mean	SD	*p-*value
F-VHI	40	0	3	0.2	0.564	0	7	0.55	1.358	0.5
P-VHI	40	0	7	0.8	1.683	0	21	1.82	4.562	0.5
E-VHI	40	0	3	0.2	0.608	0	8	0.43	1.430	1
T-VHI	40	0	10	1.2	2.564	0	36	2.8	6.944	0.5

Differences in mean scores of voice self-assessment variables before and after thyroidectomy

*SD* standard deviation, *Min* minimum observed score, *Max* maximum observed score

*p*-value—calculated probability, results of exact binomial (two-tailed) used to examine differences in the number of patients with VHI = 0 before and after thyroidectomy

The overall VHI scores before and after thyroid surgery were assigned to one of three groups (minimal, moderate or severe degree of handicap). Before thyroid surgery, all patients had the minimal degree of handicap (100%). After thyroid surgery, only one patient was handicapped, to a moderate degree (2.5%).

### V-RQOL scale

Before thyroid operations, the mean overall score (V score) for the V-RQOL scale was 99.6 with mean social-emotional domain (SE-d) 100 and physical-functional domain (PF-d) 99.2. Postoperatively, the mean V score was 98.7 with a mean SE-d 99.8 and PF-d 97.8. The binomial test revealed that the null hypothesis cannot be rejected, which means that the number of the patients who got V-RQOL score = 100 (and <100 respectively) was not significantly different before and after the treatment. This is shown in Table 3.

**Table 3:** Results of descriptive statistical analysis for voice-related quality of life before and after thyroidectomy

		Before thyroid operation				After thyroid operation				
V-RQOL score	Number of patients	Min	Max	Mean	SD	Min	Max	Mean	SD	*p-*value
Social-emotional domain	40	100	100	100	0.158	94	100	99.8	0.949	1
Physical-functional domain	40	96	100	99.2	1.176	79	100	97.8	4.802	0.5
Total	40	98	100	99.6	1.279	85	100	98.7	3.083	0.5

Differences in mean scores of V-RQOL before and after thyroid surgery

*SD* standard deviation, *Min* minimum observed score, *Max* maximum observed score

*p*-value—calculated probability, results of exact binomial (two-tailed) used to examine differences in the number of patients with V-RQOL score = 100 of voice self-assessment variables before and after thyroidectomy

The overall scores for the V-RQOL were analysed. All patients before and after thyroid operations were qualified into the 'excellent' group.

### Correlation between VHI and V-RQOL

**Fig. 1:**
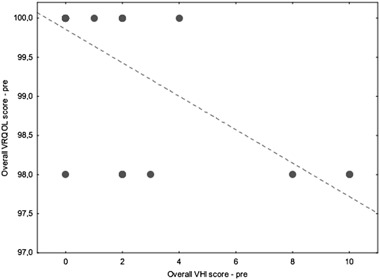
Scatter-plot showing correlation between the overall score of the V-RQOL and the overall score of the VHI scales measured before treatment. Comment: When the scores for the two questionnaires, V-RQOL and VHI, before and after treatment were compared using Spearman rho correlation test, an inverse correlation was found between the overall V-RQOL scores and the overall VHI scores (Spearman rho > 0.6, *p* < 0.001)

**Fig. 2:**
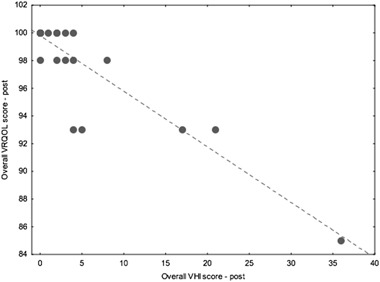
Scatter-plot showing correlation between the overall score of the V-RQOL and the overall score of the VHI scales measured after treatment. Comment: When the scores for the two questionnaires, V-RQOL and VHI, before and after treatment were compared using Spearman rho correlation test, an inverse correlation was found between the overall V-RQOL scores and the overall VHI scores (Spearman rho > 0.6, *p* < 0.001)

The scores from the VHI and V-RQOL were compared. There was a strong correlation between V-score V-RQOL and Overall score of the VHI before thyroid surgery (Spearman rho −0.68) and after thyroidectomy (Spearman rho −0.77); both *p* values < 0.001. (Figures 1 and 2).

### Correlation between VHI, V-RQOL and sociodemographic and treatment data

There was no correlation between V-scores for the V-RQOL or the O-Score of the VHI and sex, the kind of thyroid operation (primary, reoperations), diagnosis, thyroid function (euthyroidism/hyperthyroidism), the mean volume of the goitre, the presence of retrosternal position and the extent of thyroid operations (lobectomy, thyroidectomy) (Spearman R, *p* > 0.05). We found only a small correlation between the mean age of the patients and postoperative the O-Score of VHI (Spearman R, *p* = 0.007650) and between the mean age and postoperative V-Score for the V-RQOL (Spearman R, *p* = 0.00648).

## Discussion

Voice complications after thyroid surgery have been repeatedly evaluated in the literature [6–8, 16]. Despite many years since thyroid surgery was first considered quite safe, altered voice is still a problem for both patients and surgeons. About one in ten patients experiences temporary laryngeal nerve injury after surgery with longer lasting problems in up to 25% of cases. Previous data have shown that 25% to almost 90% of patients report abnormal voice in the first period after the operation and 11–15% have permanent voice problems in the first 6 months postoperatively [6, 16]. Recurrent laryngeal nerve injuries are the main, but not the only factor influencing voice quality of life after the operations. Other less common causes of voice impairment include strap muscle injury, oedema or airway management [17]. Moreover, it is worth emphasizing that temporary hoarseness happens in any surgery with general anaesthesia, but in the case of thyroid surgery it always arouses suspicion of RLN injuries [18]. Neuromonitoring distinguishes RLN damage due to surgery from anaesthetic, but it is not possible to distinguish other subtle reasons for voice changes. We excluded patients from the study with difficult intubation or oedema after intubation. Thus, early, appropriate recognition of voice problems after thyroidectomy is beneficial for patients in order to be able to get the right treatment.

In order to improve voice outcome after thyroid surgery, it is strongly recommended that a surgeon identify RLN injuries during the operation [6]. Most surgeons consider this to be the gold standard, which minimizes the rate of RLN injuries [6, 19, 20]. The next milestone in thyroid surgery seems to be the introduction of IONM during the operation. Nowadays IONM is a common technique which is gaining more and more acceptance among surgeons as an adjunct to the routine of visual recurrent laryngeal nerve injury identification [7]. With the application of IONM, the rate of RLN injury identification is almost 100% [21–24] and the incidence of transient RLN palsies is lower, particularly in difficult thyroid operations [21, 25, 26]. The introduction of IONM also focused our interest on the EBSLN [8]. So far, more attention has usually been paid to the consequences of damage to the RLN than to EBSLN injury, which is less clearly defined, but could lead to temporary or permanent voice change as well. It is an injury which causes the dysfunction of the CTM, resulting in changes in voice quality and projection, and reduction of the ability to produce high frequency tones. Some patients may also have a hoarse breathy voice with increased throat clearing and vocal fatigue [6, 8, 16]. Although for the majority of patients, a change of voice dependent on EBSLN could be discreet or even overlooked, it is a significant disability to professional voice users. Until now, the problem of injuries of the EBSLN has been neglected, because the diagnosis of its injury is difficult to confirm in solely postoperative laryngoscopy. Only laryngeal electromyography makes it possible to diagnose nerve injury and to evaluate it with high diagnostic certainty [6, 8]. Visual identification of EBSLN is usually possible in only 80% of patients undergoing routine dissection and in almost 100% with the application of IONM [27].

In the literature, there are many publications about voice assessment and voice-related quality of life in patients after thyroid operations [6, 16, 17, 28–30] but only a few of them refer to surgery with IONM [31, 32].

The aim of this study was to assess thyroid operations with IONM in the context of voice quality after surgical treatment. For this purpose, we evaluated vocal quality preoperatively and postoperatively. Among a number of existing voice-specific symptom scales, we chose two of the most commonly used instruments: Voice Handicap Index (VHI) and V-RQOL. The VHI is an instrument that was developed to quantify the patient’s perceptions of disability due to vocal dysfunction. The V-RQOL questionnaire is a self-administered short form patient report instrument which measures the subjective burden elicited by voice disorders. Both questionnaires have proved to have good reliability and validity for a range of voice disorders [11, 33].

To precisely assess even subtle changes of voice after thyroidectomies, all the patients had a thorough postoperative laryngoscopy. Our study shows that the mean functional, physical, emotional and total scores before and after thyroidectomy were different, but the differences were not statistically significant (0.2 vs. 0.55, *p* = 0.5; 0.8 vs. 1.82, *p* = 0.5; 0.2 vs. 0.43, *p* = 1; 1.2 vs. 2.8, *p* = 0.5). Similarly, there were no statistical differences between the mean V-score for V-RQOL before and after surgery (99.6 vs. 98.7, *p* = 0.5). We did not observe any statistically significant differences in the socio-emotional or physical functioning domain (100 vs. 99.8, *p* = 1; 99.2 vs. 97.8, *p* = 0.5). The biggest change between the preoperative and postoperative period concerned the physical or physical functioning domains in both questionnaires. A very strong correlation between VHI and V-RQOL before (−0.68 Spearman R) and after surgery (−0.77 Spearman) was found in the group of patients. It should be emphasized that almost all the patients after thyroid operations were handicapped to a minimal extent according to the VHI scores and have an 'excellent' V-score of the V-RQOL. Our data show that voice changes after thyroid surgery with the utilization of IONM are subtle, almost imperceptible to the patients. It seems that EBSLN identification and preservation has a significant impact on voice quality. It is very difficult to distinguish surgical reasons vs. non-surgical ones for voice changes without the use of IONM. Our study confirms that thyroid surgery with IONM, with positive responses from RLN and EBSLN during the operations, is on the top of quality treatment. Our results are similar to those observed by Randolph [30] and Su-jin Kim [29] during thyroid surgery with IONM. Randolph et al. assessed the quality of the voice after thyroidectomy with the use of IONM in a professional singer. The voice handicap index mean scores, pre-op and post-op were unchanged: VHI 4.15 (±5.22) vs. 4.04 (±3.85), *p* = 0.9301. Subsequent tests (Singing Voice Handicap Index—SVHI and Evaluation of Ability to Sing Easily) confirmed the same results as well. The Su-jin Kim study also shows very good results in voice quality after EBSLN identification. This author used IONM to identify EBSLN injury during bilateral axilla‒breast approach robotic thyroid surgery. VHI-10 showed a change of voice over time (0.1 vs. 3.6 vs. 1.3), however, this was not statistically significant. These authors also emphasize the role of IONM in the preservation of EBSLN and its influence on the quality of voice.

In the literature there are a lot of publications about the quality of voice after thyroid surgery before the introduction of neuromonitoring. In Cvelbar’s study, significant differences were found between the mean scores on voice self-assessment variables before and after thyroidectomy. These differences were found on functional, physical and total VHI score (F-VHI, P-VHI, T-VHI) [30]. Voice and swallowing changes after thyroidectomy in patients without inferior laryngeal nerve injuries were assessed in a Lombardi publication. The mean postoperative voice impairment scores (VIS) were significantly higher (*p* = 0.05) than the preoperative VIS 1 week and 1 month after thyroidectomy, but not after 3 months. EBSLN was not assessed in these publications [34]. This may be a reason why the voice-related quality of life differs significantly in pre-op and post-op. This observation confirms the beneficial effect of IONM on improving voice quality after surgery.

In our study, we tried to find significant factors influencing VHI and V-RQOL in patients who underwent thyroid surgery. Only age was found to be a significant predictor of voice quality with higher voice handicap in the younger patients. It was weak correlation, but it surprised us. This may be related to the impact of vocal dysfunction on the working and social life of younger patients. We did not find any correlation between other socio-demographic data, the diagnosis and the kind of thyroid operation.

At the end it is worth mentioning that the authors are aware of several limitations of the study. The first is the small group size of patients enrolled ino the study. The 40 patient group is small to strongly support the well-justified results. Secondly, the voice alteration might be the result of several other factors (i.e., strap muscles injury or intubation) which are difficult to distinguish from voice problems associated only with the thyroid surgery.

However, the lack of research on this subject prompted us to summarize our data and the review of current literature on the subject. Since the IONM is more and more accepted in many countries, not only the rate of RLN or EBSLN injuries, but also the voice quality of life should be taken into consideration.

## Conclusion

The use of neuromonitoring in thyroid surgery is beneficial for patients to help safeguard against loss of voice quality. The identification and preservation of EBSLN seems to be crucial to eliminate altered voice after thyroidectomy. VHI and V-RQOL are simple, easy to use and are comparable measures about the voice after thyroid operation. This gives us important and useful information about physical, functional and emotional voice outcomes after thyroidectomy.

## References

[1] Agency for Healthcare Research and Quality. Statistical brief 86. Healthcare Cost and Utilization Project (HCUP) (2010), http://www.hcup-us.ahrq.gov/reports/statbriefs/sb86.jsp. Accessed 4 June 2012 21413206

[2] Gharib H, Papini E, Valcavi R, Baskin HJ, Crescenzi A, Dottorini ME, Duick DS, Giglielmi R, Hamilton CR, Zeiger MA, Zini M (2006). American Association of Clinical Endocrinologists and Associazione Medici Endocrinologi medical guidelines for clinical practice for the diagnosis and management of thyroid nodules. Endocr. Pract..

[3] Rayes N, Steinmuler T, Schroder S, Klotzer A, Bertram H, Denecke T, Neuhaus P, Seehofer D (2013). Bilateral subtotal thyroidectomy versus hemithyroidectomy plus subtotal resection (Dunhill procedure) for benign goiter: long-term results of a prospective, randomized study. World J. Surg..

[4] Dralle H, Stang A, Sekulla C, Rusner C, Lorenz K, Machens A (2014). Surgery for benign goiter in Germany: fewer operations, changed resectional strategy, fewer complications. Chirurg.

[5] Barczyński M, Konturek A, Stopa M, Cichoń S, Richter P, Nowak W (2011). Total thyroidectomy for benign thyroid disease: is it really worthwhile?. Ann. Surg..

[6] Chandrasekhar SS, Randolph GW, Seidman MD, Rosenfeld RM, Angelos P, Barkmeier-Kraemer J, Benninger MS, Blumin JH, Dennis G, Hanks J, Haymart MR, Kloos RT, Seals B, Schreibstein JM, Thomas MA, Waddington C, Warren B, Robertson PJ (2013). Clinical practice guideline: improving voice outcomes after thyroid surgery. Otolaryngol. Head. Neck Surg..

[7] Randolph GW, Dralle H, International Nerve Monitoring Study Group (2006). Electrophysiologic recurrent laryngeal nerve monitoring during thyroid and parathyroid surgery: International Standards Guideline Statement. Laryngoscope.

[8] Barczyński M, Randolph GW, Cernea CR, Dralle H, Dionigi G, Alesina PF, Mihai R, Finck C, Lombardi D, Hartl DM, Miyauchi A, Serpell J, Snyder S, Volpi E, Woodson G, Kraimps JL, Hisham AN (2013). External branch of the superior laryngeal nerve monitoring during thyroid and parathyroid surgery: International Neural Monitoring Study Group standards guideline statement. Laryngoscope.

[9] Bergenfelz A, Jansson S, Kristoffersson A, Martensson H, Reinher E, Wallin G, Lausen I (2008). Complications to thyroid surgery: results as reported in a database from a multicenter audit comprising 3,660 patients. Lange. Arch. Surg..

[10] Jeannon JP, Orabi AA, Bruch GA, Abdalsalam HA, Simo R (2009). Diagnosis of recurrent laryngeal nerve palsy after thyroidectomy: a systematic review. Diagnosis of recurrent laryngeal nerve palsy after thyroidectomy: a systematic review. Int J. Clin. Pract..

[11] Hogikyan ND, Sethuraman G (1999). Validation of an instrument to measure voice-related quality of life (V-RQOL). J. Voice.

[12] Sielska-Badurek E, Rzepakowska A, Sobol M, Sobol-Wójcikiewicz E, Niemczyk K (2016). Adaptation and validation of the Voice-Related Quality of Life measure Into Polish. J. Voice.

[13] Jacobson BH, Johnson A, Grywalski C, Silbergleit A, Jacobson G, Benninger MS, Newman CW (1997). The Voice Handicap Index (VHI): development and validation. Am. J. Speech Lang. Pathol..

[14] Tiple C, Drugan T, Dinescu FV, Muresan R, Chirila M, Cosgerea M (2016). The impact of vocal rehabilitation on quality of life and voice handicap in patients with total laryngectomy. J. Res Med Sci..

[15] Pruszewicz A, Obrebowski A, Wiskirska-Woznica B, Wojnowski W (2004). Complex voice assessment: Polish version of the Voice Handicap Index (VHI). Otolaryngol. Pol..

[16] Meek P, Carding PN, Howard DH, Lennard TW (2008). Voice change following thyroid and parathyroid surgery. J. Voice.

[17] Soylu L, Ozbas S, Uslu HY, Kocak S (2007). The evaluation of the causes of subjective voice disturbances after thyroid surgery. Am. J. Surg..

[18] Mendels EJ, Brunings JW, Hamaekers AE, Stokroos RJ, Kremer B, Baijens LW (2012). Adverse laryngeal effects following short-term general anesthesia: a systematic review. Arch. Otolaryngol. Head. Neck Surg..

[19] Jatzko GR, Lisborg PH, Muller MG, Wette VM (1994). Recurrent nerve palsy after thyroid operations-principal nerve identification and a literature review. Surgery.

[20] Munks S (2005). Prevention of recurrent laryngeal nerve paralysis by demonstration of the nerve during thyroid surgery. Laryngorhinootologie.

[21] Barczyński M, Konturek A, Cichoń S (2009). Randomized clinical trial of visualization versus neuromonitoring of recurrent laryngeal nerves during thyroidectomy. Br. J. Surg..

[22] Dionigi G, Barczyński M, Chiang FY, Dralle H, Duran Poveda M, Iacobone I, Lombardi CP, Materazzi G, Mihai R, Randolp GW, Sitges-Serra A (2010). Why monitor the recurrent laryngeal nerve in thyroid surgery?. J. Endocrinol. Invest..

[23] Sturgeon C, Sturgeon T, Angelos P (2009). Neuromonitoring in thyroid surgery: attitudes, usage patterns, and predictive of use among endocrine surgeons. World J. Surg..

[24] Wojtczak B, Kaliszewski K, Sutkowski K, Bolanowski M, Barczyński M (2018). A functional assessment of anatomical variants of the recurrent laryngeal nerve during thyroidectomies using neuromonitoring. Endocrine.

[25] Wojtczak B, Sutkowski K, Kaliszewski K, Głód M, Barczyński M (2017). Experience with intraoperative neuromonitoring of the recurrent laryngeal nerve improves surgical skills and outcomes of non-monitored thyroidectomy. Lange. Arch. Surg..

[26] Wojtczak B, Sutkowski K, Kaliszewski K, Barczyński M, Bolanowski M (2017). Thyroid reoperation using intraoperative neuromonitoring. Endocrine.

[27] E.A. Darr, R.P. Tufano, S. Ozdemir, D. Kamani, S. Hurwitz, G. Randolph, Superior laryngeal nerve quantitative intraoperative monitoring is possible in all thyroid surgeries. Laryngoscope (2014). 10.1002/lary.24446 10.1002/lary.24446 24115215

[28] Solomon NP, Helou LB, Henry LR, Howard RS, Coppit G, Shaha AR, Stojadinovic A (2013). Utility of the voice handicap index as an indicator of postthyroidectomy voice dysfunction. J. Voice.

[29] Pernambuco AL, De Almeida MN, Matias KG, Costa EB (2015). Voice assessment and voice-related quality of life in patients with benign thyroid disease. Otolaryngol. Head. Neck Surg..

[30] Cvelbar D, Bonetti A, Simunjak B (2016). Voice quality before and after thyroidectomy. J. Spec. Educ. Rehab..

[31] Kim SJ, Lee KE, Oh BM, Bae DS, Choi JY, Myong JP, Youn YK (2015). Intraoperative neuromonitoring of the external branch of the superior laryngeal nerve during robotic thyroid surgery: a preliminary prospective study. Ann. Surg. Treat. Res..

[32] Randolph GW, Shirtan N, Song P, Franco RJ, Kamani D, Watson G, Franco RJ, Kamani D, Woodson G (2015). Thyroidectomy in the professional Singer-neural monitored surgical outcomes. Thyroid.

[33] Portone CR, Hapner ER, McGregor L, Otto K, Johns MM (2007). Correlation of the Voice Handicap Index (VHI) and the Voice-Related Quality of Life Measure (V-RQOL). J. Voice.

[34] Lombardi CP, Rafaelli M, L. D’alatri, De Crea C, Marchese MR, Maccora D, Paludetti G, Bellantone R (2008). Video-assisted thyroidectomy significantly reduces the risk of early postthyroidectomy voice and swallowing symptoms. World J. Surg..

